# Ethics Guidelines for Immersive Journalism

**DOI:** 10.3389/frobt.2019.00028

**Published:** 2019-04-24

**Authors:** Ana Luisa Sánchez Laws, Tormod Utne

**Affiliations:** ^1^Faculty of Media, Institute for Animation and Media Production, Volda University College, Volda, Norway; ^2^Faculty of Media, Institute for Journalism, Volda University College, Volda, Norway

**Keywords:** immersive journalism, ethics guidelines, journalism codes of ethics, virtual reality, 360 degree video, press ethics bodies

## Abstract

When journalists decide to invite audiences to witness a news event “as if they were there” through immersive journalism, they acquire new responsibilities toward audiences. When audiences accept such an invitation, they also must understand the responsibilities that experiencing the news event with a much higher degree of bodily involvement entails. The main goal of this article is to discuss the elements of ethics guidelines that can address the new ethical challenges brought about by immersive journalism. We wish to investigate ethics guidelines for immersive journalism from two perspectives: existing ethical contexts, insofar as these can be understood from analyzing codes of ethics and press ethics bodies rulings, and journalists' own ethical concerns when doing immersive journalism. Through an investigation of these perspectives, we wish to propose a set of key elements that can help reshape ethics guidelines so that they better address the issues raised by immersive journalism. We investigate ethics guidelines in a select sample of codes used by organizations currently practicing immersive journalism. Selected organizations include the New York Times, Washington Post, Associated Press, Reuters, Vice News, the Guardian, Al Jazeera, El País, and the regional newspaper Sunnmørsposten in Norway. A thorough discussion of these perspectives will support our proposal for how the audience dimension can be better considered in ethics guidelines for immersive journalism, by (a) establishing methods to assess early on how technologies change ethical practice, (b) making journalists and press ethics bodies more aware of the audience dimension, including the need to consider the principle of doing no harm as also involving doing no psychological harm to audiences, and (c) establishing pathways to include news audiences as partners in the construction of ethics guidelines for immersive journalism.

## Introduction

Press ethics guidelines for immersive journalism need development. The increase in dissemination of immersive journalism stories by prominent news organizations (e.g., the Guardian, the New York Times, CNN, Aljazeera, El País and many more) makes ethical issues a pressing matter. Journalists must critically consider how they will engage users with virtual reality (VR) technologies, and which role news organizations will have in promoting guidelines for engagement with VR that preserve credibility, integrity, and accuracy.

The rhetoric around immersive journalism is that this type of journalism can give greater involvement and empathy toward news issues (De la Peña et al., 2010). However, this potential to affect audiences to the extent of making them feel emotionally distressed foreshadows future dilemmas for the users of journalism, journalists, news organizations, as well as press-ethical bodies. All these actors must consider the challenges attached to a lived/bodily experience of virtually delivered news.

We must first clarify that when speaking about new ethical dilemmas, we are referring primarily to those challenges that can arise from “true VR,” that is, virtual reality that effectively immerses the viewer in the synthetic world. One could argue that the ethical challenges raised by immersive journalism are merely a continuation of those that have already been discussed in relation to visual journalism. However, the type of immersive journalism we want to focus on is the one advocated by De la Peña, where the goal is to make newsreaders feels as if they are in the middle of the news e vent, above all by creating a full bodily experience. This emphasis on the body and on the ability to act is, we argue, the fundamental difference between visual journalism and immersive journalism.

Technically, one can distinguish between 360 video based immersive journalism (for instance, a project such as The New York Times “The Displaced”) and 3D based immersive journalism (most of De la Peña's projects as well as The Guardian's “6x9” fall under this category). Yet more than making a distinction based on the technology used, and following Cruz-Neira, one may further specify that for immersion using virtual reality to occur, a system must meet the requirements of stereoscopy, motion parallax, and the ability to interact in the environment with the full body (Cruz-Neira, 2016). True virtual reality systems are then “real-time interactive graphics with three-dimensional models,” that induce the “illusion of participation in a synthetic environment rather than observation,” are “immersive, interactive, multisensory, viewer-centered,” and try to be a “clone of physical reality” (Mazuryk and Gervautz, 1996). More than technology, what needs to be highlighted is the importance of a full bodily ability to act in the environment, since this is the cornerstone of the concept of immersive journalism.

However, producers of “VR journalism” may be guilty of exacerbating the current confusion, as many of these productions stand far from meeting the ideals of immersive journalism. It is thus fairer to place productions that rely primarily on passive observation as being part of the continuum of visual journalism, and therefore treat ethical dilemmas arising from these projects as not entirely novel. Although hoping to avoid making a categorization in terms of the reproduction technology, a number of 360 degree video productions fall in the category of “pseudo-immersive journalism,” since they only partially engage the user's body or invite to action in the immersive environment. Within the literature that has dealt with 360 degree video projects, Aitamurto discusses the way in which these projects affect existing ethical norms in visual journalism (Aitamurto, 2018). In her study, which involved interviewing journalists from leading news outlets who were using 360 degree video, she found that these journalists were confronted by two paradoxes. The first paradox was that these journalists considered 360 degree video as a more accurate way of presenting the news event, given the ability to portray the full panorama without the conventional editorial choice of framing the shot, yet precisely this lack of editorial guidance could make the user have a less accurate perception due to not knowing where to look, thereby missing important elements of the event. The second paradox was that 360 degree video was increasingly involving a series of “manipulative” choices that journalists considered appropriate to safeguard authenticity. Amongst them, removing themselves from the panoramic view. Complaints about manipulation and lack of facticity have been raised about how journalists have sought to remove their presence in a 360 degree video immersive scene, often for the sake of aesthetics, by erasing tripods, by hiding, and by asking subjects to re-enact their actions after the 360 degree camera has been placed in an appropriate position. One example is the case of “The Displaced” by the New York Times, where the journalists “staged” a scene with a child riding his bicycle. The New York Times responded to criticism to this project in a column in The Public Editor by Margaret Sullivan (2015) by saying that the New York Times was aware of the many pitfalls that experimenting with VR in news would bring, and that one should consider that it took decades to develop ethics guidelines for photojournalism. The case is interesting because it points to a disconnect between current guidelines and innovative practices in journalism such as the use of immersive technologies: as will be discussed later in this article, the New York Times ethics guidelines clearly state that no photography should be staged, directed or an environment or element of a scene modified.

For Aitamurto, who builds on issues about representation discussed by cultural theorists such as Stuart Hall, these paradoxes mean that visual journalism in its 360 degree form is (again) confronting the problem of denotation vs. connotation, that is, the problem of claiming to represent something “as is” vs. showing it “as if.” Aitamurto briefly discusses these paradoxes in relation to the problem of concrete ethical guidelines, pointing out how the guidelines of the Society of Professional Journalists and the National Press Photographers Association, both in the U.S., stress the objectivity norm in visual journalism by disavowing any form of image manipulation outside of minor adjustments. Importantly, Aitamurto notes that at heart, the issue is one of being able to differentiate between visual journalism and advertising or propaganda, and this has to do with the aspirations of journalists to above all convey events as truthfully as possible.

In this article, however, we are concerned with ethical dilemmas that arise from true immersive journalism products, and we argue that while many issues related to visual journalism are shared, important dilemmas posed by immersive journalism as a form of journalism that focuses on bodily response are significantly different from those of visual journalism. Our concern stems from the potential of experiencing an intentionally misleading and fabricated perspective during an immersive experience, and how this may affect behavior in the real world. Madary and Metzinger (2016) argue that the potential for deep behavioral manipulation with immersive technologies is high, that these media are substantially different from other media in this regard, and that the influence of experiences in the virtual environment upon behavior in the real world could be significant. They cite examples of risks and effects of the possibilities virtual environments give users to transform their appearance, which is shown by phenomena that Yee and Bailenson (2007) have called the *Proteus Effect*. In two experiments, Yee and Bailenson found that people began acting according to changes in their virtual appearance, for example becoming more extrovert and outgoing when given more attractive avatars, or more confident when given taller avatars. The researchers measured these effects within the virtual environment, so these results only apply to changes in behavior during a virtual immersion. However, other experiments have dealt with how virtual reality experiences influence real world behavior. Hershfield et al. (2011) found that persons experiencing an immersive environment where their avatars looked older were more prone to allocate money to pensions in the real world after the experience, and Rosenberg et al. (2013) reported that subjects who were allowed to fly through a city like Superman in a virtual environment exhibited altruistic behavior in the real world after the immersion was over. As Madary and Metzinger discuss, these experiments are revealing how immersive experiences can have strong influence in our subsequent behavior. With examples such as The New York Times “Take Flight” (2015) immersive experience, where the audience could fly above Manhattan while interacting with famous movie stars, the concerns raised by Rosenberg et al. should not seem alien to the work of journalists. As with clothing and makeup in the real world, our appearance in an immersion will have an impact in how we behave, both within and outside the virtual environment. This is an area of high concern, given the way in which other media have been historically used for manipulation, such as film for propaganda by the Nazis in the 1930s, and more recently social media and the internet to influence elections and disrupt the democratic process.

As will be shown in the analysis that follows, current ethics guidelines in many news organizations practicing immersive journalism include a concern for the safety and wellbeing of informants as well as journalists themselves, yet guidelines on duties toward audiences are often vague. Given the above concerns related to VR technologies in general and given the lack of guidelines that consider the risks posed by news consumption in VR to news audiences, the main goal of this article will be to discuss the new responsibilities journalists and news organizations acquire toward audiences when placing them in the middle of the news event by virtual means, and furthermore, to problematize the idea of audiences as passive receptors who are free from the burden of assuming an ethical responsibility when consuming the news.

The article begins with an overview of the current state of press ethics guidelines in a selection of news media organizations that have regularly produced immersive journalism projects in the last 2 years. We must note that this paper does not intend to review a wide representative sample of general ethics codes, but rather assess a sample of codes in use by organizations currently practicing immersive journalism. We have chosen what we consider are the main innovators in immersive journalism production at this moment in time.

From this general view, we narrow down on the specific case of newspapers in Norway. We begin by discussing the general press ethics context that Norwegian newspapers operate in, by a look at the Norwegian Code of Ethics for Journalism and the work of the Norwegian Press Ethics Council. Our intention is to identify the current provisions and practices that can be applied to immersive journalism products. It is precisely at this stage, when bodies such as PFU receive complaints, that media houses have to face how to solve ethical issues that were at first not thought, and that most problems and concerns arise for editors and journalists. We therefore go into depth into describing potential issues for immersive journalism in what concerns ethics bodies rulings, using as example the PFU.

We then move on to present the experiences and ethical concerns of journalists themselves, which we gathered through interviews with journalists in three Norwegian newspapers. Our interviews show that informants were highly aware of the importance of addressing ethical concerns in their immersive journalism projects yet believed that a period of trial and error with a degree of risk taking was a necessary part of understanding how to engage with immersive journalism. We note that at the time of writing, the journalists own positions have been in constant shift from project to project, because in fact these professionals are trying to identify and pin down what may be the ethical issues these technologies bring about. It seems thus premature to us to discuss the journalist's personal position beyond the issues raised by the projects they discuss, given that these novel forms of storytelling are too young for such own position to be consolidated in any way. Our discussion is also aimed more at a systemic more than at a personal level.

Building on the above sections, we propose in the final section of the article a set of key elements for future-oriented ethics guidelines where the challenges that immersive journalism presents are more forcefully addressed.

Our two-pronged approach to the topic of ethics guidelines, namely an investigation of press ethics contexts and journalistic practices is informed by Ward's (2008) argument that journalism ethics should be about both the macro level of the role of media in society and the micro level of what individual journalists ought to do in specific situations. It is also informed by Ward's views of the need for journalism ethics that can encompass both the global and local conditions of practice. We find Ward's call for global journalism ethics especially relevant for immersive journalism, where the products released can be consumed by audiences all over the world.

## Brief Comparison of Ethics Guidelines From Organizations Using Immersive Journalism

The first step in our analysis involves a comparison of ethics guidelines of news organizations from around the globe that have been actively producing immersive journalism content in the last two years (mid 2016–2018). An effort has been made to include organizations that can represent diverse geographical regions, diverse organization types, and diverse audience demographics. Selected organizations include the New York Times, Washington Post, Associated Press, Reuters, Vice News, the Guardian, Al Jazeera, and El País. While many of these organizations have roots in print, these organizations have increasingly moved toward delivering news through a host of different formats and in a multiplicity of platforms. It is therefore interesting to assess the extent to which their guidelines are keeping up with these changes, and moreover, whether organizations that have a stronger tradition of working with video material and broadcast, such as for example Associate Press and Al Jazeera, display more awareness of potential ethical issues with immersive journalism in their guidelines. We also analyzed the ethics guidelines of Norwegian newspapers but will discuss these in more detail later in the article.

We examined the guidelines for each organization for themes that were recurrent across them. We found that the majority of ethics guidelines consulted abstain from referring to specific technologies. Rather, these guidelines attempt to function for any presentation format. However, although most guidelines avoid being too specific about technologies, the topic of photography is treated in detail across a number of them. Ethics guidelines for visual imagery are highly relevant for immersive journalism, for which we will go into further detail about the current content of the various guidelines.

### Guidelines for Image Manipulation

The Guardian's guidelines for images explicitly state that “digitally enhanced or altered images, montages and illustrations should be clearly labeled as such,” and details in images should be avoided if they can result in the identification of a location and thereby the intrusion upon the privacy or safety of subjects (The Guardian, 2007). Likewise, the New York Times “Guidelines on Integrity” state that images “must be genuine in every way” (New York Times, 2008). No people or objects may be added, rearranged, reversed, distorted, or removed from a scene,” although cropping to remove distractions is named as an acceptable and established practice. The New York Times allows alterations of color or grayscale as long as they help enhance accuracy, and images used for illustration should be appropriately labeled as such. Similar guidelines are established by Reuters in the document “A brief guide to standards, photoshop and captions,” where it is stated that journalists should never alter a still or moving image beyond normal image enhancement requirements (Reuters, 2008). The Reuters guidelines for image editing in Photoshop are very specific, stating that there should be no additions, deletions or alterations of tonal color or balance that could mislead a viewer, and that any Reuters employee conducting such alterations will be dismissed. Reuters also instructs photographers to never stage or re-enact an event, direct subjects, or remove objects. Further to that, Reuters highlights that photojournalists should be aware of how their presence may alter people's behavior. Even a misleading suggestion of association via juxtaposition of elements in an image is something Reuter warns against. When presenting guidelines for captions, Reuters stresses that captions should not provide assumptions of what a person may be thinking or feeling, but rather just present what is factually known about the image's subject. Captions should also help the public know when the subject of an image is posing for the camera. The Associated Press (AP) is also clear about avoiding fabrication in photographic and audiovisual material. AP guidelines state that “(w)e don't stage or re-enact events for the camera or microphone, and we don't use sound effects or substitute video or audio from one event to another. We do not “cheat” sound by adding audio to embellish or fabricate an event” (Associated Press, 2018).

While guidelines about image manipulation are consistent across organizations, Carlson has pointed out the dilemmas of such a stringent image regime in photojournalism (Carlson, 2009). Carlson studied the case of Brian Walski, a war photographer working for Los Angeles Times, who lost his job in 2003 due to the manipulation of a photo from the Iraq war where he combined two images taken moments apart to produce a third, aesthetically better, yet “ethically fraudulent,” third image of the fight in the field at Basra. Carlson discussed the reaction to Walski's work within the North American tradition of objective reporting, where balance, distance, neutrality, and autonomy are the hallmarks of an ethical practice. Carlson noted, however, the irony of how photojournalism is fray with instances where photographers are accused of staging or creating a photograph, yet when the images transcend their status as indexes of reality and become iconic of the events portrayed (for instance, some of Cappa's, Brady's, and Rosenthal's work), these accusations become less relevant. Carlson thus frames his argument around the idea that there is a craft inherent to photojournalism that should be better understood. He argues that perhaps we should consider that the aesthetics of the image are an essential element in a photojournalist's quest to communicate a news event in a way that can produce greater understanding.

Yet as we shall see later in our discussion of specific cases of immersive journalism, the normative boundaries to which both Aitamurto and Carlson refer to when dealing with visual journalism seem to be less relevant to journalists working with more immersive experiences (such as the case of Mysteriet i Plaza discussed later in this article).

### Guidelines Concerning the Journalist's Duty Toward Audiences

The next step in our analysis was to assess the chosen ethics guidelines in terms of how they consider journalists' duties toward audiences. We found that concrete guidelines about the responsibility toward audiences tend to be either non-existent or of a very general character. In general, the statements support the idea of the public's right to accurate information. Some guidelines indicate what kinds of audiences the news organization intends to serve, where some organizations will make their responsibility as global providers of information explicit. For example, Reuters clearly places itself as a “stateless,” global news service whose credibility relies on being seen as independent. Al Jazeera also starts its guidelines by stating that it is “a globally oriented media service,” for which reason it will treat audiences with respect, consider the feelings of victims and their families, and “recognize diversity in human societies with all their races, cultures, and beliefs and their values and intrinsic individualities so as to present unbiased and faithful reflection of them” (Al Jazeera, 2014). The Washington Post has a combined national/global approach, stating that “(t)he newspaper shall tell ALL the truth so far as it can learn it, concerning the important affairs of America and the world” and that “(w)hat it prints shall be fit reading for the young as well as for the old. The newspaper's duty is to its readers and to the public at large, and not to the private interests of the owner” (The Washington Post, 2016). More concretely in terms of the relationship to an individual newsreader, the Washington Post stresses fairness as an ethical guiding principle, stating that “No story is fair if it consciously or unconsciously misleads or even deceives the reader. Fairness includes honesty—leveling with the reader.” In the same manner, in the Editorial Standards for NYTLive, the New York Times declares that it will seek to treat audiences, as well as interviewees, speakers, and advertisers, fairly and openly (New York Times, 2015). Yet this is the point at which most guidelines stop: news organizations declare that they have a duty to inform audiences fairly. In general, no further discussion of the ways in which a news item may harm a member of the audience is made. However, certain guidelines have provisions that were originally intended for the subjects of the news can also be applied to the users of the news. This is the case for the Spanish newspaper El País, which operates within the Deontological Code of Ethics of the Federación de Asociaciones de Periodistas de España (FAPE, the Federation of Associations of Journalists of Spain). This code makes several provisions to safeguard individuals in a more inclusive fashion, as it is possible to apply these guidelines both to the subjects of news and to the users of news. For instance, the code states in point 4 that while acknowledging the public's right to information, journalists must respect a person's right to their own intimacy and self-image, and in point 6, that all the precautions regarding right to respectful treatment by the media are especially important in relation to children, in particular in cases of crime or cases that may affect their privacy (Federación de Asociaciones de Periodistas de España, 2017).

### Guidelines Concerning Audiences' Own Responsibilities as Consumers of News

Lastly, we were also interested in whether guidelines assigned any level of ethical responsibility upon audiences themselves. In general, such types of guidelines were not found. One exception was Vice News, which has a Code of Ethics that is specific to Cyber Media. As such, it distinguishes itself from other guidelines by placing an important level of responsibility on the users of the news. This takes place in section The Role of Press Ethics Bodies: The Case of the Norwegian Press Council, regarding User Generated Content, where Vice News demands that users identify themselves and agree to Vices guidelines on type of content. Vice keeps the right to delete or edit any user generated content that contains errors within 2 × 24 h yet assumes responsibility for user generated content that gets published after this period, and thus becomes in charge of making any subsequent corrections (Vice, 2012).

### Summary of Findings

To summarize the above, guidelines are very specific when it comes to the treatment of visual imagery in their approach to factuality and are very vague when it comes to the relationship between journalists and audiences. Implied in this is a certain assumption that the journalist is the sole responsible for the delivery in form and content of appropriate information to the public, which perhaps explains why what is most discussed is an issue such as form (e.g., the visuals). While our sample was mostly composed of newspapers, we have also found this to be the case in the two organizations that have a longer tradition of delivering broadcast video content (Associated Press and Al Jazeera).

It must be noted that a more stringent ethical regime may be found in publicly funded broadcasters. For example, the BBC states in its Editorial Guidelines that when representing death or events that cause suffering and distress, consideration must be given both to the impact upon victims as well as upon audiences. “Graphic scenes of grief are unlikely to offend or distress those victims and relatives who consented to our recording them, but they may upset or anger some of our audience” (BBC, 2018). The BBC recommends that the issue may be solved by presenting a contextualization that can prevent misunderstanding the scene. However, such a measure may fall short when the user is in fact asked to not only watch but also take the position of the victim. Nevertheless, the more general provisions from the BBC make room for awareness of the potential ways in which delivery of distressful news may cause ethical concerns related to audiences that will vary from platform to platform: “the use of violent images in news and documentaries require fine judgements which take account of audience expectations of content they are likely to see. Such expectations are informed by the context in which the images are used—including the nature of the output, scheduling, and the editorial purpose served by the images” (BBC, 2018).

The following table presents an overview of our findings:

**Table:** 

**Guidelines for image manipulation**	**Guidelines for journalist's duty toward audiences**	**Guidelines for audiences' own responsibility as consumers of news**
No people or objects may be added, rearranged, reversed, distorted, or removed from a scene	Global news services respect diversity and should not discriminate on the basis of ethnicity, age, gender, culture	Few outlets have such guidelines
Not acceptable to stage a scene	Journalists should not mislead audiences	Users must identify themselves online and abide to the news outlet's guidelines for content
Alterations of color or grayscale acceptable if they enhance accuracy	Both the subjects of news and news audiences should be treated fairly and openly	The news outlet assumes responsibility for user-generated content after a given period, and is in charge of making corrections
Illustrations should be labeled as such	People have the right to their own self-image, privacy, and intimacy (this is intended for subjects of news but may also apply to audiences)	
Labels should not suggest someone's emotions or feelings	Children in particular should be protected from harm	
Different images, video, or audio should not be combined to produce a new one (no synthesis)	Audience expectations and sensibilities should be taken into account	

Bjerke (2011) has problematized the way journalists assign themselves rights to the way in which they treat and inform the audience. He identifies as the social mission of journalism as (a) giving the citizens the necessary information to take reflected choices (both political and other), (b) presenting accurate information in which all parties will be heard (balanced reporting), and (c) acting open to readers, which entails avoiding double roles and bindings. This, however, also implies that journalists take for themselves the main social responsibility of informing the public accurately. Bjerke questions however the way in which this responsibility has been taken. Who has decided that the journalists should have the monopoly over factual information? One can see the lack of mention of the role of audiences in the communication and use of the news as a not entirely legitimate assumption of power in the relationship (Bjerke, 2011).

Many of these power assumptions are also expressed in the decisions of Press Ethics bodies. To examine this issue more closely, we choose in the next section to look at the specific types of decisions that can express this unbalance in the relationship between journalist and audience. Looking at Press Ethics Council's decisions is a useful way to examine how guidelines have practical consequences for journalism products and is an avenue to anticipate the issues that will be encountered in immersive journalism. Instead of providing an overview, we wish to go in the next section into the specific case of Norway, so as to give the reader a more detailed analysis of how guidelines are actually used in the field.

## The Role of Press Ethics Bodies: the Case of the Norwegian Press Council

In Norway, disputes concerning journalism ethics are resolved by the Norwegian Press Council (Pressens Faglige Utvalg, PFU), a self-governing body created by the Norwegian Press Association (Norsk Presseforbund) consisting of seven members (four members of the press and three members from the general public), which has as goal to “monitor and promote the ethical and professional standard in the Norwegian press” (Norsk presseforbund, 2018). The work of PFU is grounded on the Norwegian Ethical Code of Practice for the Press (Vær Varsom-plakaten, VVP), which was first adopted in 1936, and whose latest revision dates from 2015. We chose to go in detail into discussing this code of ethics in this section because it is the direct context for the cases of regional newspapers in Norway which will be the focus of the next section.

In brief, the VVP provides general guidelines for the role of press in society, integrity and credibility, journalistic conduct and relations with sources, and publication rules. We will limit our discussion to items of the VVP that are relevant to the issues raised by immersive journalism. The VVP contains points intended to protect journalists and the subjects of journalism, and one may argue that it is entirely based on traditional press coverage. As per the codes of ethics discussed in the previous section, some items of the VVP cover the use of visual imagery, which is a relevant topic for the highly visual media used in immersive journalism. For example, paragraph 4.11 deals with visual imagery, and has as goal to protect the credibility of the journalistic photography. It establishes that “photos used as documentation must not be altered in a way that creates a false impression. Manipulated photos can only be accepted as illustrations if it is evident that it in actual fact is a picture collage” (Pressens Faglige Utvalg, 2018). This paragraph may be used in an immersive journalism context, yet it is definitely apparent that it was not formulated for immersive journalism, where the very question of creating a false impression is exponentially complicated by the way the first-person experience can be manipulated, for example through unconscious bodily cues, as has been pointed out in the introduction through Madary and Metzinger's discussion.

One may also clearly confront conflicts about diversion or lack of boundaries between facts and fiction in point 4.2 of the VVP, which states that journalists should “make clear what is factual and what is commentary.” The intention behind this paragraph is to avoid genre blending, bringing bias and characteristics into journalistic work that is difficult to defend against for the subjects of journalism. The motive is furthermore to secure the independence and integrity of reporters. Again, the original intention behind the point has evolved from the traditional press. A potential PFU judgment on the article would probably not take into consideration the effect of immersion on the user *per se*.

No precedents exist in the form of cases brought before the Norwegian Press Council (PFU) or other European sanctions authorities concerning ethical issues related to immersive journalism. One must remember, however, that the institutionalized press ethics is always in a delay. First, journalists must gather experience, then the discourse is changed, and finally the guidelines are adjusted.

In this regard, it is important to note that general ethics guidelines such as the VVP are not the only ethical tool used by Norwegian newspapers. In-house ethical codes and editors taking the role of a devil's advocate are also common practice in a variety of news organizations in Norway. Verdens Gang (VG), a Norwegian newspaper that has become a market leader in digital news, developed their own internal ethical house rules in the early 1990's to complement the Norwegian press ethical code (VVP), because they felt they were being found guilty too many times and wanted to address the problem with more concrete internal rules. As Øy (2017) points out, several editors have followed suit: 55% of newspapers with a circulation of more than 10,000 exemplars have now separate ethical house rules, while only 17% of the newspapers with circulation under 10,000 exemplars have their own house rules.

Dalstrøm (2008) also describes the origin and distribution of in-house codes of ethics in a number of news organizations in the country. She shows how the primary focus of these codes is the journalist and her professional role, unlike the national code of ethic, which is aimed more at minimizing harmful effects of media publicity. The clear focus on roles corresponds well with the evident main task of such codes, i.e., that of strengthening the credibility of the individual media institution.

The development of in-house codes may seem to represent a lack of regulations in Norwegian newspapers, yet the self-regulating model in Norway slightly differs from other regimes. Broadcasters, especially when publicly funded, seem to be more tightly regulated, such as by the Ofcom Code in the UK. In Sweden SVT, Sveriges Radio (SR) and the Swedish Educational Radio (UR) are subject to their own ethical rules through their broadcasting licenses. A separate investigation could point out differences between ethic codes in print-originated media and broadcasting news organizations. This is nevertheless outside the scope of this paper.

In addition to general code ethics and in-house codes, Borrevik (2016) describes that it is quite common for Norwegian news organizations to consult external experts as well. Her empirical evidence shows that news organizations seek to consult the editorial association (Norsk Redaktørforening), former colleagues or editors of other publications, often relying on internal connections or connections through the country's press federations. In sum, there is a culture for seeking legal or professional advice before publishing delicate content.

As this shows, the ethical decision-making processes in a newspaper can be quite complex and are often the product of an evaluation of guidelines, internal consultation, and external advice. To better understand these processes, we asked Norwegian journalists about their views on ethical issues arising from their use of immersive journalism. We discuss some of their responses in the next section.

## Journalists' Ethical Concerns About Immersive Journalism

Our case, Sunnmørsposten, a Norwegian medium sized regional newspaper, has during the last five years achieved a position within innovative digital news storytelling in the country. With the establishment of SmpLab, an interdisciplinary unit of Sunnmørsposten consisting of coders, web designers and journalists working with data journalistic methods and tools as well as advanced multimedia storytelling techniques, the paper has produced various multimedial and immersive projects in their newsroom. As editor Hanna Relling Berg explains, Sunnmørsposten was motivated to move into immersive journalism by an urge to create new insight, new experiences and an enhanced understanding for vulnerable members of society (Relling Berg, 2018).

In 2015, Sunnmørsposten established a dialogue with the Norwegian Research Council on a project for developing multimedia long-read templates, but after a study trip to the US, the paper found that they would shift focus to developing editorial VR productions. Significant investment in VR and AR among the Silicon Valley based tech giants, as well as work done by the New York Times and Guardian, was thus the springboard for collaboration with Volda University College researchers and Teknisk Ukeblad colleagues. The goal was to make VR productions simple, effective and efficient in a way that would make them appropriate for all editors. One of the questions Sunnmørsposten wanted to ask was whether VR could be used to enhance editorial content. How could it be done, and what kinds of ethical challenges would it raise? Sunnmørsposten wanted to find out if technology could help them do an even better job and help fulfill their mission in new ways (Relling Berg, 2016).

In the process, the journalistic use of VR raised a series of questions, which the paper summarized as follows:

- “How can VR affect the user's view of journalistic material?
- What are the ethical issues journalists should pay attention to?
- Does VR contribute to a higher degree of feeling present to news events? Or the opposite, does the user feel increased distance?
- What kind of stories are best suited to immersive platforms, and what are the pros and cons?” (Relling Berg, 2016)

For SmpLab journalists, placing the user in situations that would otherwise be inaccessible was the added value of immersive journalism. One of the journalists involved in the project argued that the experience of “being there” in VR was intense, but as both the media and users matured with the use of VR, it would be easier to convey more serious journalism, which would give much greater value than flat media. Also central to the Sunnmørsposten VR initiative was the belief that an emotional and empathetic response would increase the audience's understanding of a problem (Håker Ottesen, interview with Utne, 7 November 2017).

Sunnmørsposten conducted a number of user studies during the project. Feedback from their readers after watching 360 degree video, especially feedback from a production about Loen Skylift (a gondola ride) and climbing at Slinningsbålet (a bonfire tower), pointed the newsroom in three directions. Firstly, SmpLab felt there was high entertainment value and “wow factor”: the users felt entertained and excited. Secondly, they found a value in innovation/technology, related to how 360 degree video represented a new type of experience for a large number of users. Thirdly, users expressed an improved understanding of the news item and the feeling that “now I do not have to go there myself” (Håker Ottesen, interview with Utne, 7 November 2017).

After testing the use of VR in journalism for a year, with support from Google's digital news initiative, TU journalists also found an advantage in terms of longer attention spans from readers when using rich media formats such as 360 degree video. Readers used more time to explore the 360 degree video content and therefore stayed longer on the webpage. In addition, readers reported that the technology itself was exciting to navigate in Hole (2017). In social media, the VR format has proven to be very effective in TU, as seen through very high number of interactions scores tracked through TU's social media analytics from mainly Facebook, according to Hole. The VR posts seem to have a significant wow factor and stand out from the rest of the content in their news feed. However, TU expects that time spent on reading content with 360 degree video will fall as the use of VR matures (Hole, 2017).

From the production side, journalists in Sunnmørsposten found obvious challenges in making 360 degree video within traditional media and found a need to have the format in mind from the start in planning the news coverage. Projects would have to be valued as spectacular to achieve priority. Video productions in 360 needed to be carefully planned to get thumbs up from editors. In what concerns storytelling, Håker Ottesen believes different approaches to the storytelling of VR and 360 degree video will live for a while, but the real value of VR lies in positioning users into the lead role. Traditional journalistic storytelling relies on having as main character of the story the people involved or directly affected by the news event. When using VR, how do you manage to convey the story without the face of the main character? This requires a completely different way of thinking, but Håker Ottesen thinks VR will naturally move there. The viewer will expect to play an active role, like in the gaming world. Yet these journalists also wonder if this could lead to passivity: people get the story served, it's a clear story, and they are told through imagery, text, voiceover, and music how to feel.

Thinking carefully about the ethical issues was a challenge. Journalists had to be highly conscious about how they affect the user with the new technology. “We will definitely make some mistakes that should have been avoided (…) but we are early in the race, and these are things one must learn and develop new guidelines for” (Håker Ottesen, interview with Utne, 7 November 2017). This points to the fact that in terms of ethics considerations, journalists involved in these projects had to juggle between learning a new technology and new storytelling techniques and trying to apply guidelines to their productions. In addition, working in tight deadlines and in consideration of the need of market appeal for a project to be approved was another item conditioning any kind of ethical decision-making process around a project.

Håker Ottesen believes that ethics will still be strong in journalism, but it will take time to experience immersive media, and problems will arise that should be predicted and avoided. Journalists are worried that the medium of VR will be so effective that if an ideological group wishes to portray a unilateral reality for recruiting certain people, it will be easier than today to manipulate audiences, if such groups use VR in a cynical way. One can negatively influence people to make stupid decisions, and horror movies will have a new Spring. Yet for journalism as a field, the belief is that ethics will be kept high. Editor Hanna Relling Berg believes that all forms of journalism, including VR reports, must be run in line with existing Press Ethics guidelines. “Therefore, it is crucial that time is spent clarifying the ethical issues that this technology will have to use. This is one of the reasons why Sunnmørsposten chose to use resources on the project” (Relling Berg, 2018). Another ethical challenge is also partly technical: it will be a more challenging task to go through material to capture and accurately present all angles of a situation, since 360 pictures and video require more accurate editing than media producers are used to (Håker Ottesen, interview with Utne, 7 November 2017).

To summarize, in spite of the many challenges these journalists faced, our study reveals a fairly optimistic view of the possibilities for taking immersive journalism into news rooms, according to journalists in Sunnmørsposten and Teknisk Ukeblad (TU). For them, VR evokes a higher degree of immersiveness, giving journalists opportunities they do not fully understand yet. A situation where a broader public has access to VR glasses and can participate in the moment and experience immersive content completely—from their own living room—is an exciting thought for many journalists. They feel immersive journalism has the potential to explain something that has happened in much more depth. For example, one of the journalists from SmpLab argues that with immersive journalism, people really can understand the extent of a natural disaster, and this gives journalists a whole new playroom (Håker Ottesen, interview with Utne, 7 November 2017). In addition, these journalists argue that immersive journalism can lead to greater compassion having one or more protagonists telling the story—which will produce more understanding, stronger empathy and a feeling of getting into someone else's situation in a completely different way than reading the case or watching flat video.

In the next section, we wish to go into more detail into concrete examples of practice from Norwegian immersive journalism projects where ethics guidelines are put to test.

## Examples of Ethical Challenges in Three Norwegian Immersive Journalism Products

Our first example of a concrete case where ethics guidelines are tested is from the newspaper Sunnmørsposten. In this example, the potential ethical issue concerns the clear marking of a product as advertisement or news in the aforementioned video about Loen Skylight. The 360 degree video presents a short tour up a gondola lift in a nearby town. The gondola lift video itself, as a standalone element, could be seen as advertisement. If the reader only sees this, then the product is in a frontier for what is ethically sound, in particular in relation to how it may contribute to the commercialization of a tourism actor (sections 2.6–2.8 of the VVP). If the intention of this 360 degree video had been product placement, then it should be marked clearly as such, which it is not. Yet this omission also means that Sunnmørsposten has not been paid and that the video may be unconscious advertising. However, to clarify this ethical issue further one needs to look at the context in which the immersive video was published. Sunnmørsposten wrote two journalistic articles about the gondola lift that were published in connection with the video. This way, the paper can argue that the 360 production was part of a journalistically motivated coverage. Any complaint to PFU on text advertising would hardly be accepted. The video was closely linked to the opening of a new facility, which makes it fit criteria that fully legitimate the mention of the gondola lift as a news item.

Our second example also comes from Sunnmørsposten. The paper published an immersive piece (also 360 degree video) about the prison at Ålesund, the city where the paper is located. While producing the piece, the journalist encountered a number of ethical issues associated with presenting such highly detailed depictions of the prison facility. The journalist had to make sure that scenes in the 360 degree video were presented in an order that would not reveal the actual circulation plan of the building, to prevent the video from becoming a tool for inmates wanting to escape. What seemed initially as a simple presentation of the facility became a dilemma in terms of public safety, given the possibilities that a 360 degree video could give for understanding the dimension, depth, and details of the prison.

Our third example comes from a larger Norwegian news organization, Verdens Gang (VG), with the immersive journalism project Mystery at the Oslo Plaza (“Mysteriet på Plaza”). VG wanted to investigate the Plaza case by asking: Why did nobody ever file a missing person's report on the elegant young woman found shot to death in a hotel room in Oslo? Was it homicide or suicide? Why had all personal belongings been removed from the room, and all labels cut off from her clothes? The paper wanted to present the unsolved case 20 years later and invite the audience through immersive journalism to explore crucial questions about the “Mystery at the Oslo Plaza.”

Originally the plan was even more ambitious as to be able to move around inside the room. However, this would have been too time consuming, in addition to being demanding according to what kind of equipment audiences would need to have. Thus, VG chose to land on a version that both could be experienced in web browsers and VR glasses. This made them able to render the room in a higher resolution. The entire hotel room was reconstructed in 3D, as a possible crime scene. Using VR glasses, the reader could relive the experience the investigators had when they entered the scene. In the main feature, the complicated case is told almost entirely without long text bodies. Instead, virtual reality, video and popup boxes are used. A digital long-read was available for those who wanted the complete story^1^.

One of the members of VG's editorial developer's desk was into 3D and was challenged to model the room in which the woman was found. His mission was to achieve as detailed result as possible, to be able to use it in a VR production. Some of the most challenging elements in this project involved a way to model the person and the sheets around her. This problem made the reconstruction of the victim and the hotel bed less realistic than the rest of the room. In fact, one of the first ethical issues emerged when the team working on the story had discussions on how detailed they should remake the victim. The 3D model is in theory just a model. Audiences could visualize more, and the reporters had in their possession several photos of the woman, which they decided not to use (Tom Byermoen, interview with Utne, 1–2 August 2018). For obvious reasons, VG could not publish pictures of the dead woman, but with the 3D model they still could visualize the dead woman's location in the room and what position she had in bed, which was important to understand all the strange aspects of this death. The many questions that pathologists, gun experts and court investigators have raised around the weapon's position in the hand, and the lack of crushing and blood on the hands, is thoroughly explained in text. However, the model made it easier to understand the whole situation (Lars Christian Wegner, interview with Utne, 9 August 2018).

The second ethical issue had to do with the representation of criminal violence. The Plaza mystery project was a collaboration between VG and Oslo police district. The police released all documents and photos to VG, but the images of the dead were of such a nature that they could not be published. According to the creators, when the sketch is as accurate as in this case, the journalist will approach an ethical limit at some point. The journalists landed on leaving any element reminiscent of blood out of the scene, and they discussed pixelating the victim in various ways. “Detailed images of weapons, clothes and belongings, as well as interior of the hotel room, were unproblematic, and reader reactions we received to many of these pictures have given us new, interesting information (especially about the weapon)” (Lars Christian Wegner, interview with Utne, 9 August 2018). “The purpose of presenting the case again was first and foremost to obtain new information. The hope was that someone would recognize her. At the same time, we were keen to preserve the dignity of the deceased woman” (Lars Christian Wegner, interview with Utne, 9 August 2018). All image usage from the hotel was approved by the police in advance, as it is the police who owns the scenes. The reporters made two portraits in collaboration with the police, which were assembled by different photos of the victim. This made them capable to illustrate how she probably looked alive without showing disturbing details.

When asked about responses to the image manipulation and choices made about presenting the body of the victim in Mysteriet på Plaza, the journalists at VG commented that they have so far not received any complaints from either the public or other journalists. The manipulation of the body of the victim in the simulation has thus not raised any kind of similar ethical concerns as those that could happen if the project was meant to be visual journalism.

The last ethical question came at the time of publishing the story. Originally VG planned to distribute cardboards with the print edition of the project on the day of publishing. This did not happen, partly due to ethical decisions. On the one hand, there are certain costs implied in a rural and topographically challenging country as Norway. On the other hand, a conscious decision was made to not go ahead with this idea, due to a fear that it could appear unethical to push cardboards out to the public to invite them into a crime scene.

The examples from VG and Sunnmørsposten show that ethical codes are only part of the ethical standards and practices within news organizations. Ethical decision-making is reliant on a chain of command with experienced news editors making difficult decisions at the top of this hierarchy, and in an organizational culture that places ethics at the center. This seems also to be the case internationally, as shown by Voakes (1997) for the case of American media houses, where in addition to the fear of lawsuits, factors such as in-house editorial policy and an organizational culture based on strong ethical values are essential to the enforcement of press ethics.

Moreover, ethical practice is also grounded in work distribution in the unit that will produce the immersive content. In Nordic news organizations, some news floors will be either formally structured units or be retrieved on a project basis from a news desk or similar and paired with a VR or AR programmer. Journalists rarely come straight from journalism classrooms to begin working in specialized interaction projects with programmers, web designers or other specialists. More experienced journalists are enrolled to create high quality digital journalism in interdisciplinary editorial environments. Common for those reporters is that they are well-trained journalists bringing experience with ethical issues into the multimedia units, to work together with technical competent personnel who might bring in experience from using VR in gaming and/or entertainment industries. It may take years before they qualify for a role in investigative journalism groups or in units getting to work more specialized with immersive journalism or advanced storytelling. This hierarchy and internal redeployment in news organizations will usually provide immersive projects with ethical competence.

Also, producing immersive content is a resource intensive editorial activity. Projects thus gain greater internal attention. Such high-cost content must first achieve priority, to then be subjected to both status checks and quality monitoring from editors. When media houses prioritize immersive content, this means that project leaders, editors, and executives will be following the project closely. This alertness also implies a security net in relation to ethics: high priority entails higher fear of failure, and hopefully lower risk of ethical shortcomings.

A third element that acts as prevention against ethical errors is the actual organization of the work. Much of the best of digital quality journalism in Nordic newspapers takes place in lab-based teams. For example, project descriptions and/or method reports from the Nordic Data Journalism Awards, show that the work is done by teams where reporters, developers, and designers work closely together. This interdisciplinary interaction ensures better cross-checking, where preconceived attitudes and blind zones can be challenged. The programmer may question a journalist's analysis of data sets, the designer may question a journalist's idea of the presentation, and vice versa. Environments that manage to create internal openness around the projects may benefit from an implied higher level of ethical review through such lab-based work.

## Ethics Proposals for Press Ethics Bodies, Journalists, and Audiences

As we have discussed in the previous sections, newspapers working with immersive journalism have confronted the need to make ethical decisions at every step of a project. Some of the ethical issues confronted are variations on themes that are known to the study of ethics in visual journalism, but we argue that other ethical concerns are novel and challenge the foundations of established journalism ethics. For instance, in terms of continuity with visual journalism, internalized aesthetic preferences where we are used to see what is in front of the camera but not who is behind it create interesting ethical challenges for immersive journalism. Is it truer to the news to reveal the journalist's presence? Or does this break the audiences' attention to the news event? It is acceptable in photography to crop an image, but should it be acceptable to erase a 360 degree camera tripod? However, the problem of the authenticity of 3D recreations is novel insofar as these recreations are not only to be represented, but to be bodily felt by viewers. How can journalists ensure that these representations meet editorial standards? In true immersive journalism projects, such as De la Peña's “ISPRESS,” “One Dark Night,” and “Hunger in L.A.,” The Guardian's “6x9,” and VG's “Mysteriet på Plaza,” all scenes are careful reconstructions around factual evidence. These are true synthetic worlds that claim authenticity. Such authenticity and accuracy is not claimed on the basis of realistic imagery or lack of manipulation (how could that be when everything is constructed!), and in this sense completely break with the classical concerns of visual journalism. The simulations in De la Peña's work meet the minimum requirements of visual realism, yet engage the body in a way that is credible—one feels that one is in some form of solitary confinement posture, one feels that one is walking around people in the streets of L.A. even if these streets and the humans there are low poligon renderings. In “6x9,” one can sit in the cell that is covered with graphics and effects, and still think that this may be what it feels like to be in a prison cell. And in “Mysteriet på Plaza,” there is no need to present a gruesome body covered in blood to be nevertheless able to gain valuable information about the case from the public. It seems, therefore, that the audience's trust in the immersive journalism piece may be more attached to their trust in journalists than to any claims to realism due to lack of image manipulation. In essence, the shift is one from thinking about authenticity as a problem of symbolic representation to authenticity as an issue that involves our embodied cognition. In this embodied congnition paradigm, all actants in the news event, including subjects, journalists, and audiences, will have a responsibility to acknowledge that there are more elements at play than images and their veracity, and that our actions will inevitably shape our understanding. This is a topic that we cannot develop in full in the short length of this article, but we hope that the reader may become interested in exploring the literature on virtual reality where subjects such as the role of spatial presence and agency in immersion are key (Steuer, 1992; Cummings and Bailenson, 2016; Slater and Sanchez-Vives, 2016).

Some of the questions raised by immersive journalism are as much technical as they are ethical, and we should hope that journalists will continue to dare to test these technologies as they become integrated into audience's everyday lives. To conclude our article, we wish to present in this section a proposal for what ethics guidelines for immersive journalism may look like.

First, ethics guidelines need to start including ways in which to assess early on how technologies change ethical dimensions of practice. One statement in this direction could read “we will be vigilant to how new technologies and forms of communication change the contexts of journalistic practice.” This is to say that instead of trying to be technology-neutral by making guidelines that fit any technology, news organizations need to acknowledge how technology may be challenging the very basic philosophical paradigms upon which current guidelines have been built (as Vice News does to an extent when it considers the problem of defining ethics guidelines for cyber media).

Second, press ethics bodies and journalists need to start considering the audience dimension in dispute resolutions and in ethical practice. For example, the principle of doing no harm may also need to encompass doing no psychological harm to audiences, e.g., by avoiding manipulation through contextual cues. Also, children both in the audience and as informants need to be protected. We still do not know the effect these technologies may have on young audiences.

Brurås (2016) has used a study of PFU decisions as conduit to discuss journalistic ethics at a more abstract level. We find his arguments pertinent to our article because he is concerned with a more essential aspect of journalistic ethics, namely the dominant philosophical paradigm. Brurås points out that by looking at PFU decisions, one can see that the dominant paradigm is one of “discourse ethics,” the Kantian approach which gives preference to rules and procedures. Yet other paradigms could be used, such as “virtue ethics,” the Aristotelian approach that stresses the search for “the good,” or “proximity ethics,” the care-based, empathic approach where the needs of the most vulnerable are highlighted, and where the individual's right to privacy and protection from harm is central. Brurås points out that in his study, the PFU does not make any allusions to empathy, integrity, or other journalistic traits of “virtue,” even though the PFU has ruled in favor of caring for the individual when the question has been one of pitting narrative and “publishing a good story” against protecting the subject. The main takeaway from Brurås discussion is that there seems to be a tendency to favor rules and procedures (something that one can also see reflected in guidelines for image manipulation discussed earlier). In this sense, the ethics of proximity, which is what the champions of immersive journalism advocate when they call for journalism that can generate empathy, is in contrast with traditional journalistic ethics, since it highlights subjectivity and encourages the understanding of how the journalist's emotions have a role in the covering of a story. For journalists, this could mean the approval of bias. Brurås argues that in the ethics of proximity, vulnerable individuals are given priority, which means that journalists have to play an active role in empowering the neglected. This thus seems to be the paradigm of journalistic ethics that immersive journalism practitioners advocate.

In order for press ethics bodies to start considering the challenges posed by immersive journalism, the philosophical foundation of current guidelines and decision-making processes need to be questioned from within, and Brurås insights into conceptual building blocks are thus highly useful. At base, the issue is one about the paradigm in use for the ethical system, which for the most seems to be grounded in a concern for rules and duties. While we have not conducted research in the decision-making processes of other press ethics bodies, we anticipate from what has been discussed about ethical guidelines in the previous section that other ethics bodies will also tend to operate within a “discourse ethics” framework. As long as this is the focus of guidelines and committees, it will be difficult to address in guidelines the ethical challenges that immersive journalism presents, which are much closer to what the role of an individual's emotions and senses is in morality, what Brurås describes as “proximity ethics.”

Third, ethics bodies and news organizations will need to start thinking about their audiences as partners in the construction of guidelines, and audiences themselves will need to understand that this is a responsibility they also need to take. Immersive journalism promises something unique: that one will be able to “be there.” Yet traveling to the location of the news is not a journey without perils. Journalists go through extensive training to be able to cope with the distress of reporting from conflict zones. Audiences themselves will need to start thinking that watching the suffering of others is not entertainment but involves the acknowledgment of a shared ethical responsibility. Perhaps this will be the way in which immersive journalism can contribute to rebuild the trust between news organizations and audiences, in a time when this relationship is increasingly challenged. As Stephen Ward argues, the project of an ethical journalism is everyone's responsibility (Ward, 2014).

The close partnership with the user should encourage the lowering of barriers for audience participation in the development of journalism ethics. It should be easier for the common user to report ethical concerns or specific ethical issues—both against the individual journalist or news organization, as well as through press self-governing bodies in more serious cases. For example, it should be possible that the same mechanisms newspapers use to publish that they comply with their Codes of Ethics (referring for example to their Press Ethics Bodies guidelines if someone experiences breaches) could be used for immersive journalism products, where these products can be equipped with the same type of watermark as well as an easy-to-use contact method that enables users to send queries to news organizations or to self-governing bodies. If such mechanisms are made clear, not only in the publication's colophon, but also as an interface element in every immersive journalism product, then ethical responsibility is also given to content recipients, which are now active participants in content. This participation requires a two-way interaction to a greater extent than when audiences are considered passive media consumers. Some early indications of how newspapers may be trying to incorporate these kinds of elements are seen in the Guardian's “6x9,” where a disclaimer is made at the beginning of the project which asks users to release the newspaper from the responsibility for any discomfort caused by viewing the immersive product. This is one way of dealing with the issue of who has responsibility, but our proposal is that newspapers have to go beyond only protecting themselves from liability, and use the opportunities provided by immersive journalism to establish a closer dialogue with audiences about ethical matters in journalism.

Creating an audience channel in immersive journalism projects where it is possible to send queries with ethical concerns will also signal a responsibility that distinguishes journalistic use of immersive content from the fiction-based use that is common in the gaming and entertainment industry. Such a distinction could create a positive flow of information that builds trust and credibility based on openness and communication. Such channels will help acknowledge the user perspective in the ethical work of journalists, as well as promote increased awareness of the strong power and responsibility editors bestow upon users when inviting them to experience immersive journalism.

## Ethics Statement

The empirical study described in a section of this article was carried out in accordance with the recommendations of the Norwegian Data Authority ethical guidelines for research from 2017, and meets the requirements from the new 2018 EU General Data Protection Regulation (GDPR) (Datatilsynet, 2018). The survey conducted in the study was anonymous (without IP logging or gathering of personal information), and required written informed consent from all subjects.

## Author Contributions

AS is responsible for collaboration in test design and data gathering (VR tests), statistical data analysis, theoretical framework, and article writing. TU is responsible for collaboration in test design and data gathering (interviews and VR tests) and article writing. Both authors have collaborated in developing the article's conclusions as well as revised the final submitted version of the article.

### Conflict of Interest Statement

The authors declare that the research was conducted in the absence of any commercial or financial relationships that could be construed as a potential conflict of interest.

## Footnotes

^1^https://www.vg.no/spesial/2017/plaza/
